# Coil-out: comparing outcomes after prostate artery embolization with and without endovascular coiling

**DOI:** 10.1186/s42155-026-00660-4

**Published:** 2026-02-21

**Authors:** Griffin P. J. McNamara, Matthew Carter, Lucas R. Cusumano, Justin P. McWilliams

**Affiliations:** 1 Department of Radiology, Division of Interventional Radiology, Ronald Reagan University of California, Los Angeles (UCLA), Medical Center, David Geffen School of Medicine at UCLA, 757 Westwood Plaza, Los Angeles, CA 90095 USA; 2 David Geffen School of Medicine at UCLA, 885 Tiverton Drive, Los Angeles, CA 90095 USA; 3 Present affiliation: Department of Interventional Radiology, University of WA School of Medicine University of Washington Medical Center, Montlake 1959 NE Pacific Street, 2Nd Floor, Seattle, WA 98195-7117 USA

## Abstract

This retrospective study evaluates post-procedural symptom score changes after prostate artery embolization (PAE) with (Coil-out) and without (Standard) adjunctive prostate artery coiling after particle embolization. Changes in IPSS, QOL/Bother, and SHIM-IIEF V are reported at 1–5- and 9–15-month intervals. 573 procedures were reviewed, and 317 patients were included with a mean age of 72.1 years. Results favored the Standard group with greater IPSS reduction at 1–5 (-12.2 vs. -9.9, *p* = 0.018) and 9–15 months (-12.5 vs. -7.6, *p* = 0.018), and greater QOL/Bother improvement in the Standard group at 9–15 months (-2.8 vs. -1.8, *p* = 0.014). Procedure times were longer in the Standard group (186 vs. 173 min; *p* = 0.039), whereas fluoroscopy times were longer in the Coil-out group (48.3 vs. 44.8 min; *p* = 0.014). Though limited by retrospective nature and lack of longer-term follow-up, these results support particle embolization alone over adjunctive coil embolization for PAE.

## Background

Lower urinary tract symptoms (LUTS) secondary to benign prostatic hyperplasia (BPH) affects three out of four men aged 60–79 and greatly reduces quality of life for patients with symptoms refractory to medical therapy [1]. As a disease which affects older patients, many present with comorbidities, which can make general anesthesia or surgical intervention difficult or unsafe. Development and optimization of minimally invasive, outpatient interventions are important for this patient population.

Prostatic artery embolization (PAE) is a well-recognized treatment for lower urinary tract symptoms from benign prostatic hyperplasia. In recent years, radiologic and urologic societies recognize PAE as a suitable alternative to transurethral or open surgical therapies such as transurethral resection of the prostate (TURP) or prostatectomy [2–6].

The first PAE was performed with polyvinyl alcohol (PVA) particles, and since then numerous protocols have been developed to include newer embolic particles or spheres, coils, n-butyl cyanoacrylate glue, or a combination of these embolic agents [7–10]. Adjunctive coil embolization after particle embolization has been touted as a way to potentially improve effectiveness and durability of PAE, however, only one retrospective non-comparative study has so far been published, which reported similar short term outcomes when compared to available literature [11].

This study examines short-term (1–5 months) and medium-term (9–15 months) outcomes for patients who received coiling after particle embolization (Coil-out) vs. particle embolization alone (Standard). By evaluating differences in outcomes after different embolization techniques, this study iterates on the evolving practice of PAE and aims to describe whether adjunctive coiling after particle embolization provides better symptomatic relief.

## Methods

**Fig. 1:**
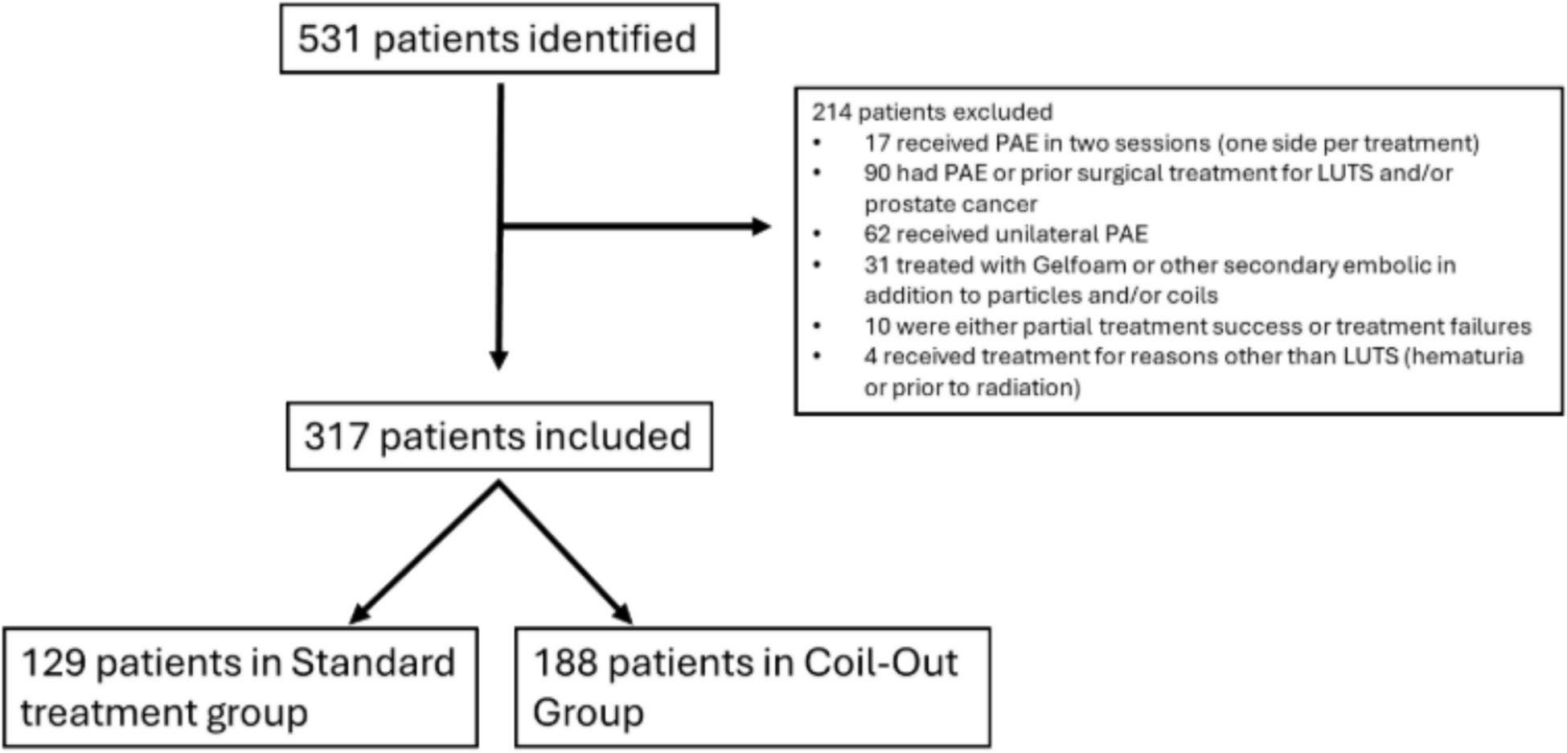
Patients excluded from study

Retrospective chart review of 573 PAE procedures between 2012 and 2025 was performed. Patients were divided into two groups, in which both prostatic arteries were coiled (Coil-out) after particle embolization, or neither of the prostatic arteries were coiled (Standard). Patients who required more than one session for PAE, had unilateral or incomplete particle embolization, had PAE for reasons other than LUTS, or had prior surgical treatment for LUTS or prostate cancer were excluded from the analysis (Fig. 1). Additionally, patients who received unilateral coiling after particle embolization were excluded. IPSS cannot be obtained in patients with indwelling Foley catheters, so these patients were described separately.

Right common femoral artery access was performed in 92% of cases. After selective catheterization of each prostatic artery, reconstituted particles were administered until a static contrast column for at least 5 heartbeats was achieved. In the coil-out group, microcoils were then placed into the prostatic arteries as an adjunctive treatment, or to achieve stasis if additional particle embolization was deemed unsafe due to collateral vasculature or tenuous catheter position.

Statistics were performed using R. Variables pertaining to demographics, treatment specifications, and symptom score change were analyzed with independent sample T tests or Mann-Whitney U tests if variables violated assumption of equality of variances and/or normality. Outcomes were reported at 1–5 months follow-up and 9–15-month follow-up based on several commonly used scoring systems (IPSS, QoL/Bother, and SHIM-IIEF V). Medication cessation was assessed at each time point by comparing baseline and follow up use of alpha antagonists (AA), and/or 5-alpha reductase inhibitors (5-ARI). Percentage of patients who are on medication at baseline who stopped at each follow up point are reported. Between-group analyses were performed with Chi-squared tests.

## Results

After IRB approval, 531 patients were identified from the 573 procedures. After exclusion, 129 patients were included in the “Coil-out” group and 188 were included in the “Standard” group (Fig. 1). Of the eight interventional radiologists who performed the procedures, one performed 66% of the cases and the top three performed 97% of cases. The Coil-out group was older (74.4 vs. 70.5; *p* < 0.001), however, there was no significant difference in baseline prostate size or median lobe grade (Table 1). Outcomes are reported as intervals from 1–5 months and 9–15 months and mean/median months to follow-up for each group are included in Table 1.

**Table 1:** Demographics, prostate characteristics based on preoperative CT angiogram, preopoerative symptom scores, and follow-up intervals in each group

*Demographics*	*Coil-out*	*Standard*	*P value*
***N***	129	188	
***Age***	74.4	70.5	<.001*
***Ethnicity (%)***			
*White/non-Hispanic*	71.3	70.4	
*White/Hispanic*	6.9	4.3	
*Black/non-Hispanic*	2.3	3.7	
*Asian*	7.0	5.9	
*Hispanic/Asian*	0.8	0	
*Other*	11.4	14.9	
**Baseline symptom scores [95% CI]**			
*IPSS*	20.4 [19.0–21.9]	20.9 [19.9–21.9]	0.62
*QOL/Bother*	4.6 [4.4–4.9]	4.7 [4.5–4.8]	0.94
*SHIM-IIEF V*	15.1 [13.6–16.7]	16.0 [14.9–17.1]	0.42
***Preop Prostate Characteristics***			
*Prostate Volume (ccs) [95% CI]*	136.6 [121.6–152.1] *N* = 89	127.1 [113.64–140.6] *N* = 128	0.09
*Median Lobe Grade* *[95% CI]*	1.5 [1.26–1.84] *N* = 42	1.67 [1.44–1.89] N = 109	0.56
***Follow up intervals***			
*1–5 month mean*	2.9	2.7	
*1–5 month median*	3	3	
*9–15 month mean*	12.1	11.9	
*9–15 month median*	12	12	

**Fig. 2:**
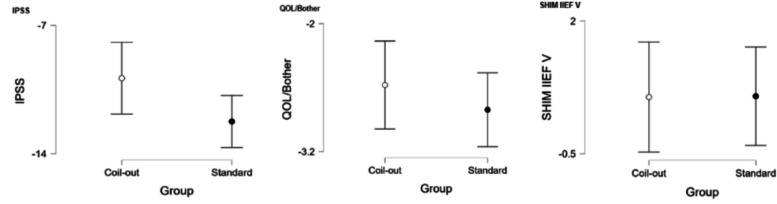
Results for all included patients at 1–5 months

**Fig. 3:**
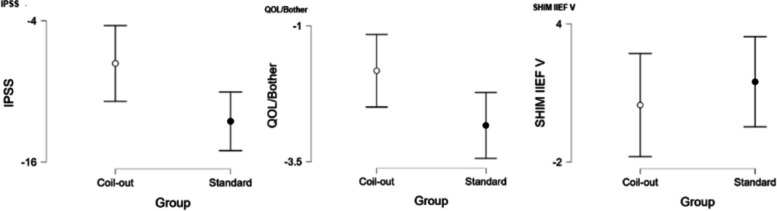
Results for all included patients at 9–15 months

The Standard group had a greater reduction in IPSS score at 1–5 months (−12.2 vs. −9.9, *p* = 0.018; Fig. 2, Table 2). Neither group had significantly different changes in QOL/Bother scores, or SHIM IIEF-V at 1–5 months (Table 2). Similarly, at 9–15 months the Standard treatment group had a greater reduction in IPSS and QOL/Bother when compared to the Coil-Out Group (IPSS: −12.5 vs. −7.6, p = 0.018; QOL/Bother: −2.8 vs. −1.8, p = 0.014), and there was no significant difference SHIM-IIEF V between groups (Fig. 3, Table 3). Of the patients with an indwelling Foley catheter and adequate clinical follow-up, 8/15 (53%) in the Coil-out group and 15/19 (79%) in the Standard treatment group were liberated from indwelling Foley. Both groups had reduced rates of medication use after treatment. At initial follow up in patients on AA and/or 5-ARI at baseline, 26% (15/57) of patients in the Coil-Out group, and 36% (30/83) of patients in the Standard group stopped all medications. At 9–15 month follow up, 36% (8/22) of Coil-Out Patients, and 52% of Standard patients were off medications for LUTS. There was no significant difference between groups at 1–5 or 9–15 month follow up (*p* = 0.27, and *p* = 0.224, respectively).

**Table 2:**
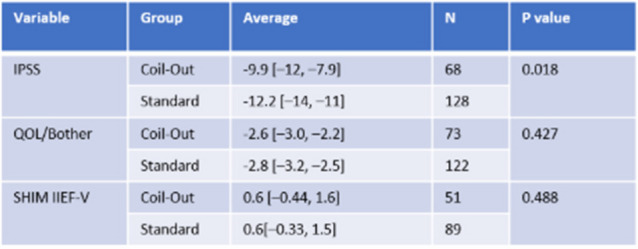
Outcomes in each group at 1–5 months

All included tests are Mann-Whitney U tests

**Table 3:**
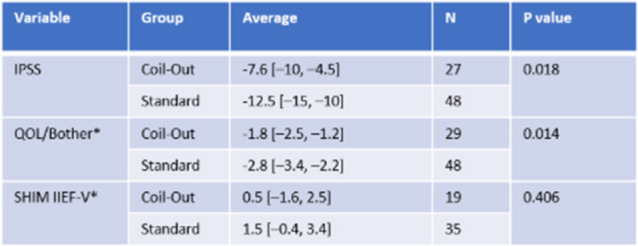
Outcomes in each group at 9–15 months

*Indicates Mann-Whitney U test. Remainder are independent sample t-test

Procedure times were longer in the Standard group (186 vs. 173; *p* = 0.039) and there were larger volumes of contrast used in the Standard group (126 vs. 115; *p* = 0.008) (Table 4). The Coil-out group had longer fluoroscopy times (48.3 vs. 44.8 min; *p* = 0.014) (Table 4). There was no significant difference in volume of reconstituted embolic between the two cohorts (Table 4). 37.9% and 42.6% of patients in the Coil-out, and Standard group, respectively, underwent PAE with the PErFecTED technique.

**Table 4:**
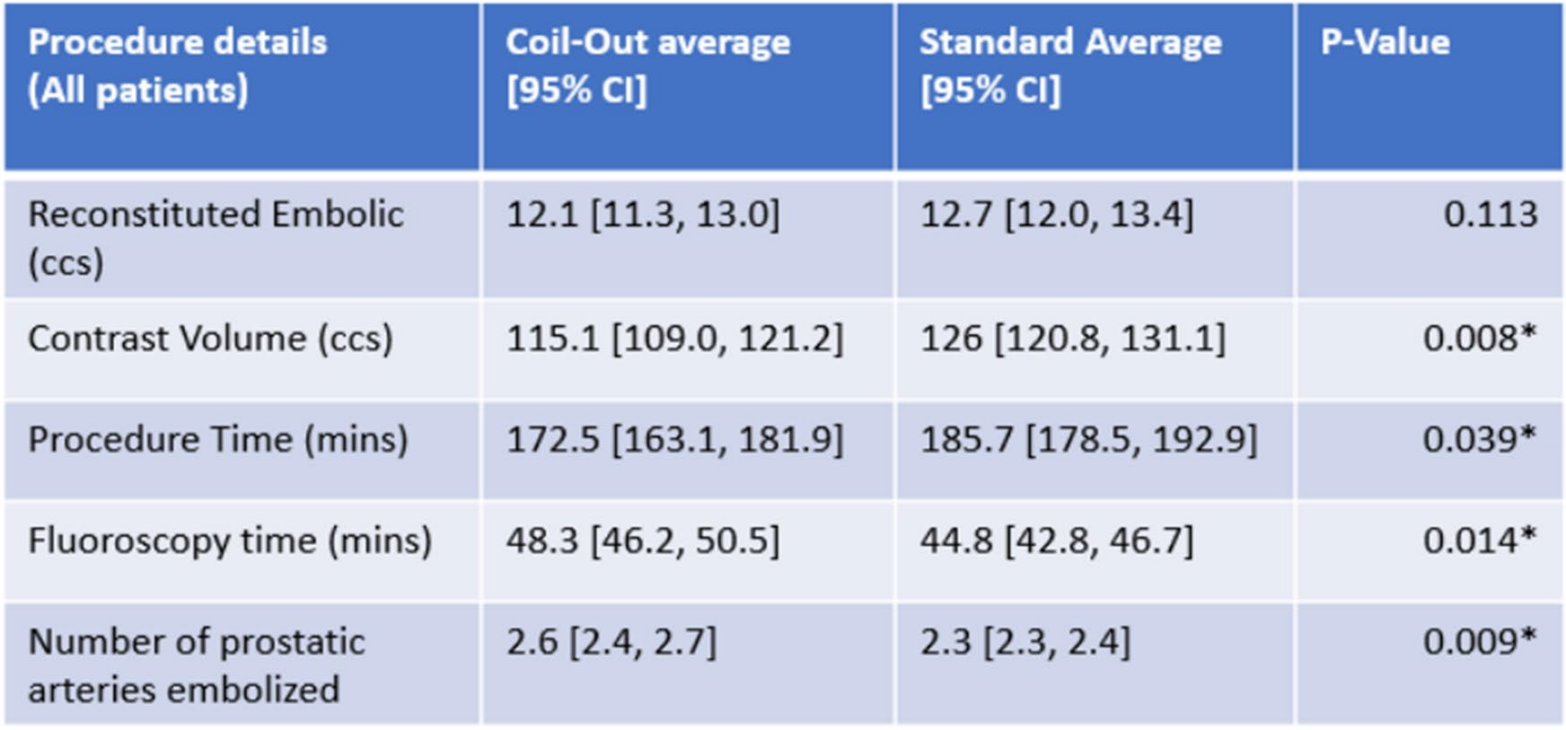
Procedure details in each group

All included results are Mann-Whitney U tests

*Indicates statistical significance

Embospheres (Merit Medical Systems, South Jordan, Utah), most commonly 300–500 micron, were used in 89% of included cases. Hydropearl (Terumo Interventional Systems, Tokyo, Japan), typically 200 (+ −75) micron, were used in 10% of cases, and 250 micron Embozene (Varian Medical Systems, Palo Alto, California) were used in 1% of cases. The majority (69%) of coils used were Ruby (Penumbra, Inc. Alameda, CA), followed by Optima/Prestige (19%) (Balt, Montmorency, France), with remaining coil systems comprising < 5% of coils used each. Approximately 1.3 coils were used per prostatic artery on average in the Coil-out group.

**Fig. 4:**
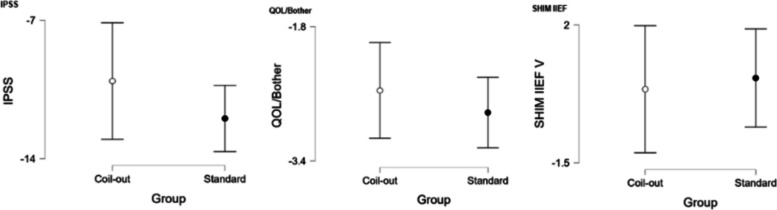
Single prostatic artery per side outcomes at 1–5 months

**Fig. 5:**
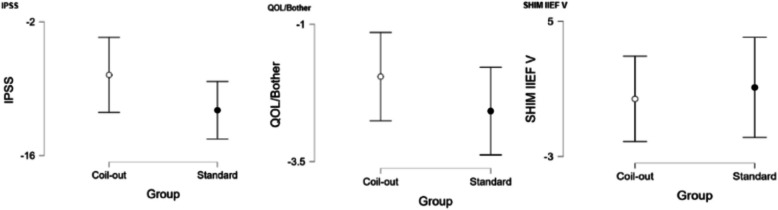
Single prostatic artery per side outcomes at 9–15 months

There were more prostatic arteries embolized per treatment in the Coil-out group (2.6 vs. 2.3; *p* = 0.009), so subgroup analysis was performed to only include patients with one prostatic artery on each side. There was no significant difference in symptomatic outcome measures between Standard or Coil-out at 1–5 months or 9–15 months in the single prostatic artery per side subgroup (Fig. 4, Table 5 and Fig. 5, Table 6). IPSS improvement trended toward significance at 1–5 months, *p* = 0.08 (Table 5). Medication cessation rates were similar across these subgroups. 27% (10/37) of Coil-Out and 38% (23/60) of Standard patients stopped medications for LUTS on initial follow up. At 9–15 month follow up, 37% (7/19) of Coil-Out patients, and 50% (12/24) of Standard patients on medication at baseline required no medications for treatment of LUTS. Again, there was no significant difference between groups at 1–5 or 9–15 month follow up (*p* = 0.254, and *p* = 0.388, respectively).

**Table 5:**
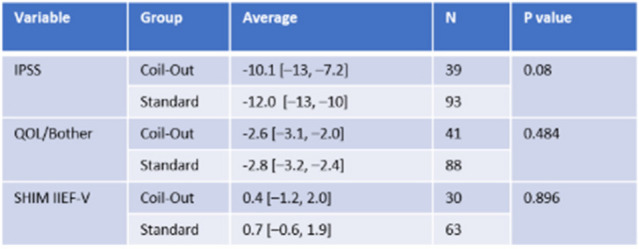
Single prostatic artery per side subgroup results at 1–5 months

All included results are Mann-Whitney U tests

**Table 6:**
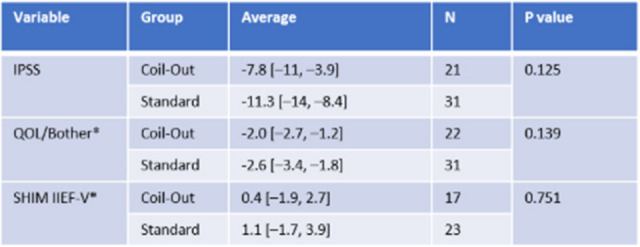
Single prostatic artery per side subgroup analysis at 9–15 months

Remainder are independent sample t-tests

*Indicates Mann Whitney U test

Nearly all patients were discharged on the same day. Eight patients in the Coil-out group and 12 in the standard treatment group underwent overnight observation. There were no major, one moderate, and three minor complications by SIR criteria [12]. The moderate adverse event was a common femoral artery pseudoaneurysm in the Coil-out group which was successfully treated with thrombin injection before discharge on post op day 1. Minor adverse events included a urinary tract infection which was treated before discharge on post-op day 3 in the Coil-out group, one patient with hematuria requiring bladder irrigation in the Standard group, and one patient with self-resolving erectile dysfunction in the Standard group.

## Discussion

Since the conception of PAE, procedural techniques have undergone constant iteration, and some operators have suggested that embolizing the prostatic arteries with coils after particle embolization may improve outcomes and durability. In this non-randomized, retrospective study, adjunctive coiling after particle embolization did not improve short-term or medium-term outcomes after PAE. Particle embolization without coiling (Standard) produced greater reduction in IPSS at 1–5 months and greater reduction in both IPSS and QOL at 9–15 months compared to particle embolization plus coiling (Coil-out). The Standard group also had a higher percentage of patients who were liberated from indwelling Foley (79% vs. 53%).

Both groups had significant symptomatic improvement. Reductions in IPSS and QOL/Bother score were similar to those reported in literature and these results add to the growing body of literature to support PAE as a safe and efficacious treatment for LUTS secondary to BPH [5, 13]. At 9–15 months, 87% (65/75) of all patients included had reduction in IPSS from baseline and 73% (55/75) had at least a −5-point change in IPSS.

Only one prior study has evaluated the use of adjunctive coiling following particle embolization for PAE [11]. In that study, the short-term outcomes with adjunctive coils were similar to the prior literature for embolization with particles only. So, the finding of the present study that adjunctive coil embolization did not improve outcomes is not particularly surprising; but the apparent inferiority of the Coil-out group was unexpected. Several reasons may account for worse-than-expected outcomes in the Coil-out group. First, the patients in this study were not randomized and the reason for “coiling out” was not recorded. While adjunctive coiling was often used after routine particle embolization because of anticipated durability benefit, adjunctive coiling was sometimes performed due to suboptimal particle embolization, such as with tenuous microcatheter position, early stasis with a low volume of particles, or concern for non-target embolization. Also, prostatic arteries which were very difficult to access were sometimes coiled to decrease the need to re-access the vessel on a repeat procedure. So, the Coil-out group may have reflected a patient group who had a less complete or more difficult PAE.

Most of the clinical and technical factors were substantially similar between the Standard and Coil-out groups. Pre-procedural symptom scores, prostate volume and median lobe grade were similar, the volume of particle embolic used was not different, and a similar percentage of patients in each group underwent the PErFecTED technique. However, patients in the Coil-out group were older, and the average number of prostatic arteries embolized in the Coil-out group was higher, which supports that PAE in the Coil-out group may have been more difficult. To limit the effect of this bias, subgroup analysis was performed to investigate the patients who had only one prostatic artery embolized on each side; this demonstrated no difference in short-term and medium-term outcomes between the two groups. Furthermore, this subgroup analysis helped to control for differences between groups by removing differences in vascular supply and prostate artery anatomy.

Procedure time and fluoroscopy time were 13 and 3.5 min shorter in the Coil-out group than the Standard group, and 10.9 cc of additional contrast was used on average in the Standard group. This may reflect a difference in interventionalist experience as most of the coil-out procedures were performed more recently than the standard procedures.

Postoperative complications were rare, and there was no clinically significant difference in complications or side effects between the two groups. Erectile function was not affected by PAE and not different between the two groups.

Causal inference of outcomes is limited due to the design of this study. The major limitations of this study include lack of prospective randomization, limited sample size, lack of long-term follow-up, absence of routine post operative imaging to assess changes in prostate volume, and lack of urine flow rate assessment on pre- and post-procedural assessment. Also, the varied reasons which determined patient selection for adjunctive coiling discussed above may have led to the inclusion of more difficult or less complete particle embolization in the Coil-out group.

These limitations notwithstanding, the present study does not support the routine use of coils as an adjunct to particle embolization for PAE. Other benefits of avoiding adjunctive coil embolization include lower procedural cost and complexity, and easier re-access of the prostatic arteries during repeat PAE.

## Data Availability

Not applicable.

## Abbreviations

PAE: Prostate artery embolization
LUTS: Lower urinary tract symptoms
TURP: Transurethral resection of the prostate
AA: Alpha Antagonist
5-ARI: 5 Alpha Reductase Inhibitor

## References

[1] Wei JT, Calhoun E, Jacobsen SJ. Urologic diseases in America project: benign prostatic hyperplasia. J Urol. 2005;173(4):1256–61. 15758764 10.1097/01.ju.0000155709.37840.fe

[2] Mirakhur A, McWilliams JP. Prostate artery embolization for benign prostatic hyperplasia: current status. Can Assoc Radiol J. 2017;68(1):84–9. 27887933 10.1016/j.carj.2016.06.003

[3] Sandhu JS, et al. Management of lower urinary tract symptoms attributed to benign prostatic hyperplasia (BPH): AUA guideline amendment 2023. J Urol. 2024;211(1):11–9. 37706750 10.1097/JU.0000000000003698

[4] McWilliams JP, et al. Society of Interventional Radiology Multisociety Consensus Position Statement on Prostatic Artery Embolization for Treatment of Lower Urinary Tract Symptoms Attributed to Benign Prostatic Hyperplasia: From the Society of Interventional Radiology, the Cardiovascular and Interventional Radiological Society of Europe, Société Française de Radiologie, and the British Society of Interventional Radiology: Endorsed by the Asia Pacific Society of Cardiovascular and Interventional Radiology, Canadian Association for Interventional Radiology, Chinese College of Interventionalists, Interventional Radiology Society of Australasia, Japanese Society of Interventional Radiology, and Korean Society of Interventional Radiology. J Vasc Interv Radiol. 2019;30(5):627-637. e1. 30926185 10.1016/j.jvir.2019.02.013

[5] Abt D, et al. Comparison of prostatic artery embolisation (PAE) versus transurethral resection of the prostate (TURP) for benign prostatic hyperplasia: randomised, open label, non-inferiority trial. BMJ. 2018. 10.1136/bmj.k2338. 29921613 10.1136/bmj.k2338 PMC6006990

[6] Carnevale FC, et al. Transurethral resection of the prostate (TURP) versus original and PErFecTED prostate artery embolization (PAE) due to benign prostatic hyperplasia (BPH): preliminary results of a single center, prospective, urodynamic-controlled analysis. Cardiovasc Intervent Radiol. 2016;39:44–52. 26506952 10.1007/s00270-015-1202-4

[7] DeMeritt JS, et al. Relief of benign prostatic hyperplasia-related bladder outlet obstruction after transarterial polyvinyl alcohol prostate embolization. J Vasc Interv Radiol. 2000;11(6):767–70. 10877424 10.1016/s1051-0443(07)61638-8

[8] Bilhim T, et al. Predictors of clinical outcome after prostate artery embolization with spherical and nonspherical polyvinyl alcohol particles in patients with benign prostatic hyperplasia. Radiology. 2016;281(1):289–300. 27223621 10.1148/radiol.2016152292

[9] Loffroy R, et al. Prostate artery embolization using n-butyl cyanoacrylate glue for urinary tract symptoms due to benign prostatic hyperplasia: a valid alternative to microparticles? J Clin Med. 2021;10(14):3161. 34300327 10.3390/jcm10143161 PMC8307138

[10] Bhatia S, et al. Role of coil embolization during prostatic artery embolization: incidence, indications, and safety profile☆. J Vasc Interv Radiol. 2017;28(5):656-664. e3. 28284886 10.1016/j.jvir.2017.01.004

[11] Galla N, et al. Adjunctive coil embolization of the prostatic arteries after particle embolization for prostatic artery embolization. Cardiovasc Intervent Radiol. 2021;44:1994–8. 34561744 10.1007/s00270-021-02964-5 PMC8475843

[12] Khalilzadeh O, et al. Proposal of a new adverse event classification system by the SIR standards of practice committee. J Vasc Interv Radiol. 2017;28(2):S153. 10.1016/j.jvir.2017.06.019 28757285

[13] Ray AF, et al. Efficacy and safety of prostate artery embolization for benign prostatic hyperplasia: an observational study and propensity-matched comparison with transurethral resection of the prostate (the UK-ROPE study). BJU Int. 2018;122(2):270–82. 29645352 10.1111/bju.14249

